# Negative Video Capsule Endoscopy Had a High Negative Predictive Value for Small Bowel Lesions, but Diagnostic Capability May Be Lower in Young Patients with Overt Bleeding

**DOI:** 10.1155/2021/8825123

**Published:** 2021-05-07

**Authors:** Sipawath Khamplod, Julajak Limsrivilai, Uayporn Kaosombatwattana, Nonthalee Pausawasdi, Phunchai Charatcharoenwitthaya, Supot Pongprasobchai, Somchai Leelakusolvong

**Affiliations:** ^1^Department of Medicine, Faculty of Medicine, Siriraj Hospital, Mahidol University, Bangkok 10700, Thailand; ^2^Division of Gastroenterology, Department of Medicine, Faculty of Medicine, Siriraj Hospital, Mahidol University, Bangkok 10700, Thailand

## Abstract

**Background:**

Patients with potential small bowel bleeding (PSBB) who have negative results of video capsule endoscopy (VCE), clinical course, rate of rebleeding, and missed lesions with their predictors are essential for further management decision.

**Methods:**

This retrospective study included patients presenting with PSBB who had negative VCE findings between January 2008 and December 2016. All patients had to have at least two years of follow-up data to be included. Patients with <2 years of follow-up in their medical record were interviewed by telephone to determine if any unrecorded rebleeding episodes occurred.

**Results:**

One hundred forty-two patients were included. The mean age was 60.9 years, and 52.8% were male. Eighty-one patients presented with overt bleeding. The median duration of follow-up was 5.08 years. During the follow-up period, 30 patients experienced rebleeding. The cumulative rate of rebleeding at 1, 2, and 5 years was 10.0%, 14.3%, and 22.4%, respectively. Multivariate analysis showed nonsteroidal anti-inflammatory drugs (NSAIDs) and presentation of overt bleeding to be independent predictors of rebleeding. There were only nine small bowel lesions (6.3%) missed by VCE. These nine patients, compared with others, were significantly younger and tended to present with overt bleeding.

**Conclusion:**

Rebleeding was not uncommon in PSBB after negative VCE; however, the rate of missing small bowel lesions was low. Nonetheless, further investigations may be considered in young patients who present with overt bleeding.

## 1. Introduction

Potential small bowel (SB) bleeding, previously defined as obscure gastrointestinal bleeding, is defined as a gastrointestinal bleeding that is undetectable by esophagogastroduodenoscopy (EGD) and colonoscopy. Video capsule endoscopy (VCE) is currently recommended as the first-line investigation in potential SB bleeding since it is relatively noninvasive and has a considerable diagnostic yield [1–4]. Positive findings can lead to diagnosis and appropriate management, while negative findings can provide high negative predictive value for rebleeding. Therefore, the European Society of Gastroenterology Endoscopy (ESGE) guideline recommends to “wait and see” if the result is negative [2]. However, there have been some reports of missed lesions, which could be detected by other investigative modalities, such as computed tomography (CT) or device-assisted enteroscopy (DAE) [5–8]. The missed lesions in these studies included various types of lesion, such as small bowel tumors, angiodysplasia, and ulcers. Delayed diagnosis particularly of a small bowel tumor might allow the tumor to progress, and this could be expected to adversely influence the prognosis. Prompt further investigations in patients with risks of missed lesions should be reasonable.

Although there have been many reports of the outcomes of patients with negative findings on VCE, limited numbers of studies included patients with a long duration of follow-up [9]. Furthermore, the number of studies that reported details specific to missed small bowel lesions is low [10–16], and none of those studies reported the factors that predict those missed lesions. Accordingly, the aim of this study was to investigate and report the rebleeding rate, the missed detection rate, and their predictors in patients with a potential small bowel bleeding and negative VCE result, who had a long duration of follow-up.

## 2. Materials and Methods

### 2.1. Study Design and Participants

This retrospective cohort study was conducted at a university-based tertiary referral center in Bangkok, Thailand. All patients who presented with potential small bowel bleeding, either overt or occult, but with no bleeding lesions on VCE between January 2008 and December 2016 were included. In patients who underwent VCE more than one time, only the first VCE was included. Patients in whom the capsule did not enter the small bowel or who had insufficient data in their medical records were excluded. This cohort included some patients in our previous study, which compared VCE and CT enterography in patients with PSBB [8], which could result in a high rate of CT enterography performed in this cohort. All patients had to have at least two years of follow-up data. Patients with <2 years of follow-up in their medical record were interviewed by telephone to determine if any unrecorded rebleeding episodes had occurred.

Patient data, including clinical information derived from medical records, laboratory data, imaging data, and procedures, were gathered from our center's institutional database. Patient characteristics, comorbidities, medications, clinical presentations, laboratory results at the time of bleeding episode, date and findings of VCE and other investigative modalities, rebleeding events, and date of last follow-up were also collected and recorded.

The protocol of study was approved by the Siriraj Institutional Review Board of the Faculty of Medicine Siriraj Hospital, Mahidol University, Bangkok, Thailand, on 12 October 2018 (COA no. 564/2561(EC4)).

## 3. Definitions

- Rebleeding was defined as any episode of passing melena and/or hematochezia or the development recurrent anemia
- VCE-missed lesion was defined as any lesion found by positive result of other investigation(s) during the first bleeding episode or any lesion subsequently found during a rebleeding episodes

### 3.1. Video Capsule Endoscopy Technique and Categorization of Findings

Video capsule endoscopy was performed using a PillCam™ SB2 video capsule (Given Diagnostic Imaging Systems, Yokneam, Israel). The fasting period began the night before the examination. Bowel cleansing was achieved with two liters of polyethylene glycol solution taken 4–12 hours before the beginning of the examination. Images were interpreted using Rapid Reader software (versions 7.0 and 8.0; Given Diagnostic Imaging Systems).

VCE images were classified into one of the three following categories: 0: negative with no identified bleeding source; P1: a lesion with intermediate bleeding potential; P2: a lesion with high bleeding potential (Supplementary Table 1) [17]. Patients with P2 lesions were excluded from the analysis.

### 3.2. Statistical Analysis

Continuous data are expressed as mean and standard deviation or median and range depending on data distribution. Categorical variables are given as frequency and percentage. Two-group comparison was performed using independent *t*-test or Mann–Whitney *U* test for continuous variables and chi-square test or Fisher's exact test for categorical variables. Cumulative probability of rebleeding was estimated using Kaplan–Meier survival curves. Cox proportional hazards regression was employed to identify significant predictors of rebleeding. A *p* value <0.05 was considered statistically significant. SAS Statistics software (SAS, Inc., Cary, North Carolina, USA) was used for all statistical analyses.

## 4. Results

One hundred and seventy-seven patients with potential small bowel bleeding who had negative findings on VCE were identified. Thirty-five patients were excluded due to having less than two years of follow-up and could not be contacted via telephone. In the end, 142 patients were included in our analysis. Demographic data, clinical characteristics, and presentations of the study population are summarized in Table 1. The mean age was 60.9 years, and 52.8% were male. Eighty-two (57%) patients presented with overt bleeding. The median duration of follow-up was 5.08 years (range: 2.2–11.7), and the total follow-up time was 808 person-years.

**Figure 1:**
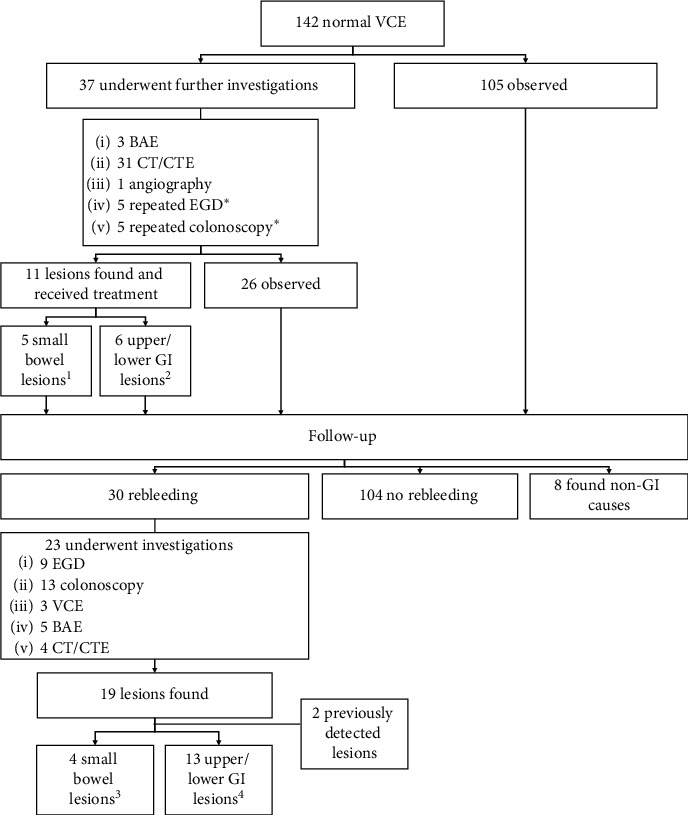
Flowchart of subsequent investigations and clinical course of study patients. *Note.∗*^∗^Repeated EGD and colonoscopy were performed in the same session of VCE. 1Five small bowel lesions included three small bowel tumors, detected by CTE, one Meckel's diverticulum, detected by CTE, and one TB ileitis, diagnosed by balloon-assisted enteroscopy with biopsy.2Six upper/lower GI lesions included a pancreatic rest, one patient with *H. pylori*-associated gastritis, one patient with colonic diverticulosis, one patient with colonic Dieulafoy's lesion, and two patients with bleeding internal hemorrhoids. All lesions except colonic Dieulafoy's lesion were found in the first EGD and colonoscopy but were not suspected to be the causes of bleeding. Definite diagnoses of these lesions were made after obtaining negative VCE results in combination with resolution of bleeding after receiving treatment^.3^Four small bowel lesions, which were discovered at a rebleeding episode, included two small bowel tumors, diagnosed by CTE, one Meckel's diverticulum, detected by balloon-assisted enteroscopy, and one small bowel angiodysplasia, detected by small bowel enteroscopy.^4^Thirteen upper/lower GI lesions, which were discovered at rebleeding episode, included one esophageal ulcer and one duodenal ulcer from upper GI sources, four colonic diverticular bleedings, one colonic Dieulafoy's lesion, one colonic telangiectasia, two colonic ulcers, including solitary rectal ulcer syndrome and ischemic ulcer, and three internal hemorrhoids from lower GI sources.

A flowchart of subsequent investigations and clinical course of study patients is shown in Figure 1. Thirty-seven patients underwent further GI investigations after VCE during the same bleeding episode, including 31 CT abdomen (either conventional or enterography technique), three balloon-assisted enteroscopy, one angiography, five repeated EGDs, and five repeated colonoscopies. From these investigations, 11 lesions were found, including five small bowel lesions, two upper GI lesions, and four lower GI lesions. The five small bowel lesions missed by VCE were as follows: three small bowel tumors, detected by CTE, one Meckel's diverticulum, detected by CTE, and one tuberculous ileitis, diagnosed by balloon-assisted enteroscopy with biopsy. The details of the missed upper and lower GI lesions are shown in Figure 1. All detected lesions were treated except one small bowel mass due to risk of operation.

**Figure 2:**
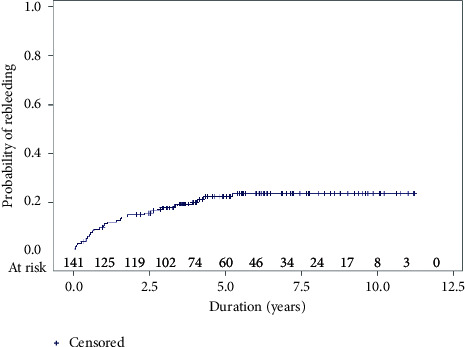
Cumulative probability of rebleeding.

During the follow-up period, 30 (21%) patients had recurrent overt bleeding or anemia. The cumulative rate of rebleeding at 1, 2, 3, and 5 years was 10.0%, 14.3%, 17.4%, and 22.4%, respectively (Figure 2). The investigations performed at the time of the rebleeding episodes are shown in Figure 1. The bleeding source was identified in 19 patients. Two patients had rebleeding from the same lesion, which was identified during the first episode of bleeding (1 small bowel mass and 1 colonic diverticular bleeding). Of the remaining 17 patients, the causes of rebleeding in the small bowel of four patients were as follows: small bowel tumors, which were detected by CTE, in two patients; angiodysplasia in one patient and Meckel's diverticulum in one patient, which were diagnosed by balloon-assisted enteroscopy. The bleeding site was detected in the upper or lower GI tract in 13 patients. The details of the detected upper and lower GI lesions are shown in Figure 1. Eight patients were found to have non-GI causes of anemia on later investigations, including myelodysplastic syndrome, anemia of chronic kidney disease, and hemoglobin H disease.

In summary, of 142 patients with potential small bowel bleeding and normal VCE in this cohort, the cause of bleeding was identified in the small bowel in nine (6.3%) patients, and lesions were found in the upper or lower GI tract in 19 (13.4%) patients. Eight (5.6%) patients were found to have non-GI causes of anemia. The cause of anemia or GI bleeding could not be definitely determined in 106 (74.7%) patients. The final diagnoses of study patients are shown in Table 2.

### 4.1. Predictive Factors for Rebleeding

As shown in Table 3, the factors for rebleeding included in the analysis were age, gender, comorbid illnesses, medications, such as NSAIDs, antiplatelets, and anticoagulants, clinical presentations, serum hemoglobin, and serum albumin. NSAIDs use and presenting with overt bleeding were significant predictors in univariate analysis. In multivariate analyses adjusted for age and gender, NSAIDs use remained significant with a hazard ratio (HR) of 6.430 (95% confidence interval [CI]: 2.111–19.584; *p*=0.0011), while overt bleeding nearly achieved the level of statistical significance to be an independent predictor of rebleeding (HR: 2.275, 95% CI: 0.998–5.184; *p*=0.0504).

### 4.2. Predictive Factors for Small Bowel Lesions Missed by VCE

Patient characteristics, comparing between those whose lesions were missed or were not missed by video capsule endoscopy, are shown in Table 4. Patients with missed lesions were significantly younger than patients with nonmissed lesion (47.1 years vs. 61.8 years, resp.; *p*=0.009). Moreover, the missed lesion group demonstrated a nonsignificant trend of presenting with more overt bleeding than the nonmissed lesions group (88.9% *vs.* 55.9%, resp.;*p*=0.078). Multivariate analysis could not be performed due to patients with a small number of missed lesions.

### 4.3. Progression of Patients with Uncertain Diagnosis and Effect of Antiplatelets/Anticoagulants Discontinuation

Among 106 patients with uncertain source of bleeding, 44 patients took antithrombotic agents (antiplatelets and/or anticoagulants), including 20 patients taking single antiplatelet, eight patients taking single anticoagulant, six patients taking dual antiplatelets, nine patients taking single antiplatelet plus single anticoagulant, and one patient taking dual antiplatelets plus single anticoagulant. Of 44 patients taking antithrombotic agents, nine patients discontinued all agents, 10 patients discontinued some agents, and 25 patients temporarily discontinued the medications and later resumed taking the same agents as before bleeding.

**Figure 3:**
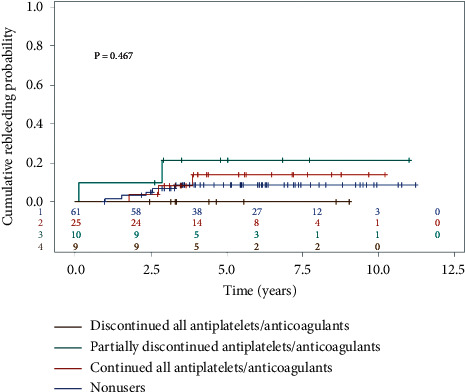
Cumulative rebleeding probability of patients with uncertain diagnosis classifying by status of antithrombotic agents.

During follow-up, the rebleeding rates were 0% in patients discontinuing all antithrombotic agents, 20% in those partially discontinuing the medications, 12% in those continuing taking the same medications, and 8.1% in those never taking antiplatelets or anticoagulants. There was no significant difference in cumulative rebleeding probability among groups as shown in Figure 3 (*p*=0.467). Ninety-four patients had hemoglobin values available at the follow-up period (nine patients discontinuing all antithrombotic agents, 10 patients partially discontinuing the medications, 22 patients continuing taking the same medications, and 53 patients never taking antiplatelets or anticoagulants). The hemoglobin levels at baseline and at last follow-up of the patients in each group are shown in Table 5. All four groups had significant improvement of hemoglobin levels at the last follow-up when compared with their baseline hemoglobin levels (paired *t*-test *p* < 0.01 in all groups). Patients who completely discontinued antithrombotic agents appeared to have a higher mean hemoglobin level, higher proportion of patients with normal hemoglobin levels, and lower proportion of patients with severe anemia at the last follow-up when compared with the patients who continued taking the same antithrombotic agents, but they were not statistically different.

## 5. Discussion

ESGE guideline 2015 recommends conservative management in patients with PSBB who do not have ongoing bleeding. [2] The results of our study are consistent with this recommendation as we showed that VCE had a high negative predictive value for small bowel lesions. However, some missed small bowel lesions were observed and the missed rate was comparable to previous studies [10–16]. Therefore, further investigations may be required in some patients. To identify patients who may require further investigations, predictive factors for the missed small bowel lesions are needed.

Our study included 142 patients, and all patients had more than two years of follow-up (median follow-up duration: 5 years). Rebleeding was found in 21% of patients, which is comparable with the proportion reported from a 2017 meta-analysis [9]. In the present study, a significant number of rebleeding episodes occurred during the first two years. The cumulative rate of rebleeding at one and two years was 10.0% and 14.3%, respectively. The corresponding values in previous studies were 12.9%–27% and 25.6%–32.1%, respectively [11, 13, 15]. These results suggest that careful monitoring is needed during the first two years after negative VCE. The independent predictors for rebleeding in our study were overt GI bleeding and use of NSAIDs. Overt GI bleeding was also reported as a risk factor for rebleeding from univariate analysis in other reports [13, 15, 20]. Another independent risk factor was NSAIDs use. The overall rate of overt GI rebleeding was reported to be 20.4% within two years among NSAIDs users [21]. NSAIDs are an over-the-counter family of drugs that are widely available in Thailand, so the risk of medication resumption is high. Notably, among 19 rebleeding patients with detected lesions, 13 (68.4%) lesions were found outside the small bowel. This bleeding rate from outside of small bowel is comparable to that reported by Harada et al., who found that eight of thirteen (61.5%) patients with rebleeding had bleeding from outside the small bowel [15].

Regarding lesions located in the small bowel that were missed by VCE, there were only nine lesions in nine patients (6.3%). The most common type of missed lesions was small bowel tumor (5 patients), followed by Meckel's diverticulum (2 patients). The most useful investigation that could complement VCE in this study was CT enterography, which could detect five tumors and one Meckel's diverticulum. The rate of small bowel lesions missed by VCE in our study is comparable with other studies, which reported rates ranging from 2.4% to 9.0% [10–16]. The types of lesions missed by VCE varied among studies. In contrast to our cohort, angiodysplasia and ulcers were the major lesions that were missed by VCE in the studies by Matsumura et al. and Magalhães-Costa et al. [12, 13]. We believe that this variation between and among studies is attributable to not only differences in study populations but also the frequency and type of additional diagnostic investigations. Cross-sectional imaging was most often used in our study, while Matsumura et al. most commonly used DAE. Small bowel tumors have been reported to be missed by VCE but detected by CT enterography in some studies [22, 23]. Balloon-assisted enteroscopy possibly detects any type of lesions, including mucosal lesions, if they can be reached. The potential predictors for missing lesions by VCE were young age and presenting with overt bleeding. This may be explained by the fact that the majority of lesions missed were small bowel tumors and Meckel's diverticulum, and these are the most common causes of potential small bowel bleeding in the young [1].

Optimizing antiplatelets and anticoagulants is crucial. These agents could induce bleeding from tiny lesions that could have been missed by VCE, such as small angiodysplasia [24]. Continuing anticoagulation therapy has been reported to be associated with rebleeding in patients with potential small bowel bleeding, who had negative VCE [10, 11]. In this study, among patients with uncertain diagnosis and who took either antiplatelets or anticoagulants or both, those who completely discontinued these agents had no rebleeding during the follow-up period. Furthermore, nearly half of them had normal hemoglobin levels, and none had severe anemia at the last follow-up. This improvement seemed to be better than the patients who did not adjust antithrombotic agents where the rebleeding rate was 12.0%, only 27.3% had normal hemoglobin levels, and 9.1% had severe anemia at the last follow-up. Although the difference is not statistically significant, it is possibly because of inadequate power, and this data should support that optimizing use of antithrombotic agents is mandatory in this group of patients.

### 5.1. Strengths and Limitations

The strength of this study is that we included a large number of patients compared with the populations enrolled in previous studies. The other important strength is that we included only patients with at least two years of follow-up. There are some limitations of our study. First, our study is of a retrospective approach. Management of each patient was based on their treating physicians. Second, since this was a single-center study that was conducted in a tertiary referral center, certain biases could have exerted some adverse influences, such as a bias towards more complicated patients, which were referred from other centers. The factors that were found to be associated with rebleeding after negative VCE and with missed lesions in this study should be validated in a large multicenter prospective study.

## 6. Conclusion

The present study demonstrates that, in potential small bowel bleeding with a negative VCE examination, observational management is advisable; however, patients should be carefully followed up, particularly during the first two years, since there is a possibility of rebleeding of about 20%. Moreover, the discontinuation of NSAIDs and optimization of antithrombotic agents should be emphasized if bleeding recurs, and bleeding from outside the small bowel should also be suspected. The rate of bleeding from the small bowel was low but should be considered in young patients with overt bleeding due to the higher possibility of the presence of small bowel tumors or Meckel's diverticulum. In these cases, CT enterography may be considered.

## Figures and Tables

**Table 1:** Baseline characteristics of patients in this cohort.

Characteristics	*N* = 142
Age (year), mean ± SD	60.9 ± 16.5
Male gender, *n* (%)	75 (52.8%)
Diabetes, *n* (%)	36 (25.4%)
Atherosclerosis^∗^*∗*, *n* (%)	45 (31.7%)
Chronic kidney disease†, *n* (%)	32 (22.5%)
(i) Stage 3	17 (12.0%)
(ii) Stage 4	2 (1.4%)
(iii) Stage 5	13 (9.1%)
Presence of colonic diverticulosis, *n* (%)	26 (18.8%)
NSAIDs, *n* (%)	4 (2.9%)
Antiplatelets, *n* (%)	47 (33.8%)
Anticoagulants, *n* (%)	22 (15.7%)
Overt bleeding, *n* (%)	81 (57.0%)
Presenting duration (day), median (range)	46 (0–1,925)
Abdominal pain, *n* (%)	9 (6.4%)
Weight loss, *n* (%)	5 (3.6%)
Hemoglobin level (g/dL), mean ± SD	8.58 ± 2.48
Albumin level (g/dL), mean ± SD	3.64 ± 0.67
Follow-up time in years, median (range)	5.08 (2.20–11.70)

*∗* ^∗^Atherosclerosis included any coronary artery, cerebrovascular, or peripheral vascular disease. ^†^Chronic kidney disease was defined according to Kidney Disease: Improving Global Outcomes (KDIGO) definition, which was glomerular filtration rate less than 60 mL/min/1.73 m^2^ for at least three months. [18]. SD: standard deviation; NSAIDs: nonsteroidal anti-inflammatory drugs.

**Table 2:** Final diagnosis of study patients (*n* = 142).

Diagnosis	*n* (%)
Small bowel mass	5 (3.5%)
Small bowel telangiectasia	1 (0.7%)
Small bowel *tuberculosis*	1 (0.7%)
Meckel's diverticulum	2 (1.4%)
Upper or lower GI source	19 (13.4%)
Non-GI causes	8 (5.6%%)
Uncertain diagnosis	106 (74.7%)

GI: gastrointestinal.

**Table 3:** Univariate and multivariate analyses for factors significantly associated with rebleeding after negative video capsule endoscopy.

Factors	Univariate HR (95% CI)	*p*	Multivariate HR (95% CI)	*p*
Age	1.004 (0.982–1.027)	0.707	1.007 (0.985–1.029)	—
Male gender	2.151 (0.985–4.698)	0.054	1.501 (0.660–3.414)	—
Diabetes	1.025 (0.456–2.305)	0.952	—	—
Atherosclerosis^∗^*∗*	0.936 (0.429–2.045)	0.869	—	—
Chronic kidney disease	0.559 (0.195–1.604)	0.280	—	—
Colonic diverticulosis	1.318 (0.562–3.088)	0.526	—	—
NSAIDs use	7.254 (2.495–21.088)	0.0003	6.430 (2.111–19.584)	0.0011
Antiplatelet use	0.721 (0.319–1.627)	0.430	—	—
Anticoagulant use	1.375 (0.562–3.366)	0.486	—	—
Overt bleeding	2.350 (1.045–5.281)	*0.039*	2.275 (0.998–5.184)	0.0504
Duration of presentation	1.000 (0.998–1.001)	0.790	—	—
Abdominal pain	0.532 (0.072–3.908)	0.535	—	—
Weight loss	0.847 (0.115–6.220)	0.871	—	—
Hemoglobin level	0.957 (0.823–1.113)	0.567	—	—
Albumin level	0.831 (0.416–1.658)	0.599	—	—

*∗* ^∗^Atherosclerosis included any coronary artery, cerebrovascular, or peripheral vascular disease. HR: hazard ratio; CI: confidence interval; NSAIDs: nonsteroidal anti-inflammatory drugs.

**Table 4:** Characteristics of patients whose lesions were missed or were not missed by video capsule endoscopy.

Characteristics	Missed by VCE (*n* = 9)	Not missed by VCE (*n* = 133)	*p*
Age (year), mean ± SD	47.1 ± 17.7	61.8 ± 16.1	0.009
Male gender, *n* (%)	7 (77.8%)	68 (51.1%)	0.121
Diabetes, *n* (%)	0 (0.0%)	36 (27.1%)	0.112
Atherosclerosis, *n* (%)	2 (22.2%)	43 (32.3%)	0.719
Chronic kidney disease, *n* (%)	1 (11.1%)	31 (23.3%)	0.683
NSAIDs, *n* (%)	1 (11.1%)	3 (2.3%)	0.237
Antiplatelets, *n* (%)	2 (22.2%)	45 (34.6%)	0.718
Anticoagulants, *n* (%)	1 (11.1%)	21 (16.0%)	>0.99
Overt bleeding, *n* (%)	8 (88.9%)	73 (55.9%)	0.078
Presenting duration (day), median (range)	14 (0–323)	47 (0–1,925)	0.390
Abdominal pain, *n* (%)	1 (11.1%)	8 (6.1%)	0.458
Weight loss, *n* (%)	0 (0.0%)	5 (3.8%)	>0.99
Hemoglobin level (g/dL), mean ± SD	7.99 ± 1.83	8.62 ± 2.52	0.463
Albumin level (g/dL), mean ± SD	3.78 ± 0.54	3.63 ± 0.68	0.600

Data are shown in *n* (%), unless specified. A *p* value <0.05 indicates statistical significance. VCE: video capsule endoscopy; SD: standard deviation; NSAIDs: nonsteroidal anti-inflammatory drugs.

**Table 5:** Hemoglobin levels at baseline and last follow-up among patients with uncertain diagnosis classifying by status of antithrombotic agents.

	Antiplatelets or anticoagulants usage				
	Totally discontinuing (*n* = 9)	Partially discontinuing (*n* = 10)	Continuing (*n* = 22)	Nonusers (*n* = 53)	*p*
Hb at baseline (g/dL)	9.00 ± 1.89	8.09 ± 1.80	7.63 ± 2.20	9.09 ± 2.90	0.14
Hb at last follow-up (g/dL)	11.51 ± 1.89	11.16 ± 2.08	10.84 ± 2.27	11.67 ± 2.16	0.48

*Anemia severity* ^∗^ *∗*					
(i) Normal (hb ≥ 12 g/dL)	4 (44.5%)	3 (30.0%)	6 (27.3%)	27 (51.0%)	0.43
(ii) Mild (hb 10–11.9 g/dL)	2 (22.2%)	4 (40.0%)	9 (40.9%)	15 (28.3%)	
(iii) Moderate (hb 8–9.9 g/dL)	3 (33.3%)	3 (30.0%)	5 (22.7%)	6 (11.3%)	
(iv) Severe (hb < 8 g/dL)	0 (0%)	0 (0%)	2 (9.1%)	5 (9.4%)	

*∗* ^∗^Adapted from World Health Organization. Hemoglobin concentrations for the diagnosis of anemia and assessment of severity [19]. Hb: hemoglobin.

## Data Availability

The data supporting the conclusions of the study are available upon request to the corresponding author.

## Supplementary Materials

Supplementary Materials Supplementary Table 1: diagnostic categories used for video capsule endoscopy.
