# Corrigendum to “Negative Video Capsule Endoscopy Had a High Negative Predictive Value for Small Bowel Lesions, but Diagnostic Capability May Be Lower in Young Patients with Overt Bleeding”

**DOI:** 10.1155/cjgh/9836801

**Published:** 2025-02-04

**Authors:** Sipawath Khamplod, Julajak Limsrivilai, Uayporn Kaosombatwattana, Nonthalee Pausawasdi, Phunchai Charatcharoenwitthaya, Supot Pongprasobchai, Somchai Leelakusolvong

**Affiliations:** ^1^Department of Medicine, Faculty of Medicine, Siriraj Hospital, Mahidol University, Bangkok 10700, Thailand; ^2^Division of Gastroenterology, Department of Medicine, Faculty of Medicine, Siriraj Hospital, Mahidol University, Bangkok 10700, Thailand

In the article titled “Negative Video Capsule Endoscopy Had a High Negative Predictive Value for Small Bowel Lesions, but Diagnostic Capability May Be Lower in Young Patients with Overt Bleeding” [1], there was an error in the Materials and Methods section. This error is shown below:

“COA no. 564/2561(EC4)” should read “COA no. Si 643/2018.”
