# Supplementary material for: Negative Video Capsule Endoscopy Had a High Negative Predictive Value for Small Bowel Lesions, but Diagnostic Capability May Be Lower in Young Patients with Overt Bleeding
Source: Can J Gastroenterol Hepatol. 2021 May 7;2021:8825123. doi: 10.1155/2021/8825123 (PMC8123999; doi:10.1155/2021/8825123)
Supplement: Supplementary Materials — Supplementary Table 1: diagnostic categories used for video capsule endoscopy. [file 8825123.f1.docx]

**Supplementary Table 1.** Diagnostic categories used for video capsule endoscopy

|  | **Negative (P0)** | **Intermediate bleeding potential (P1)** | **Lesion with high bleeding potential (P2)** |
| --- | --- | --- | --- |
| **Video capsule endoscopy** | Negative | Red spot  Small erosion | Inflammatory lesion (Ulcer/enteritis)  Angiodysplasia  Tumor  Diverticulum  Bleeding without identifiable source |
